# Establishment of an efficient in vitro propagation protocol for Sumac (*Rhus coriaria* L.) and confirmation of the genetic homogeneity

**DOI:** 10.1038/s41598-020-80550-4

**Published:** 2021-01-08

**Authors:** Saleh Amiri, Reza Mohammadi

**Affiliations:** Branch for Northwest and West Region, Agricultural Biotechnology Research Institute of Iran (ABRII), Agricultural Research, Education and Extension Organization (AREEO), Tabriz, Iran

**Keywords:** Biotechnology, Plant sciences

## Abstract

The conventional reproduction methods are not efficient for regeneration of Sumac (*Rhus coriaria* L.). The purpose of this work was to study the micropropagation of *R. coriaria* using lateral buds as explant in Murashige and Skoog (MS) medium with different concentrations of plant growth regulator (PGRs). Four concentrations of Benzylaminopurine (BAP) in combination with three concentrations of indol-3-butyric acid (IBA) and 1.0 mg/L gibberellic acid (GA3) were tested for establishment and shoot multiplication. For root induction, IBA was used at four levels combined with 0, 0.5 and 1 mg/L of naphthalene acetic acid (NAA) in full and half strength of MS medium. BAP at 2 mg/L with 1 mg/L IBA was best, with 88.88% of establishment. The highest shoot proliferation (12.30 ± 0.30) was obtained in medium fortified with 2 mg/L BAP plus 0.5 mg/L IBA and the highest shoot length (8.50 cm) was obtained at 3 mg/L BAP plus 1 mg/L IBA. The highest rooting (100%) was observed in 1/2-strength MS medium containing 1 mg/L IBA with 0.5 mg/L NAA. In conclusion, an efficient protocol with high rate of proliferation and rooting is described for *R. coriaria*, which can be used in massive propagation.

## Introduction

Sumac (*Rhus coriaria* L.) is a shrub belonging to Anacardiaceae family, which includes around 81 genera and more than 800 species. It is widely spread in subtropical and temperate areas throughout the world, especially in Africa, Iran, South Eastern Anatolia, the Mediterranean area, and Western Asia^1^. *R. coriaria* has been used in human nutrition (as a drink, appetizer, sauce, natural acidulate in food recipes, dried fruits powder as spice). It is also used in traditional medicine for treating eye diseases, wounds, bowel disorders, ringworms, skin disorders, anorexia, diarrhea, hemorrhage, and hyperglycemia, astringent agent and antimicrobial activities^1–4^. Iranian sumac, with ornamental properties like small spreading canopy, multi-trunk structure, stunning brilliant autumn color, and low requirements for water and nutrients, could be a good candidate for urban landscapes, particularly in climates similar to Mediterranean regions^5^. On the other hand, sumac is important in environment conservation in slopes. Sumac planting is one of the farmer's sustainable strategies for soil and water conservation on the slopes of Hurand region, East Azarbaijan, Iran, where this plant also has a critical role in rural farmer’s life and family income. Sumac is a suitable species for the revegetation of degraded land in the Mediterranean region. Because of its importance, demand for sumac rootstocks is increasing, then mass propagation is needed^6^. In current horticultural practice, *R. coriaria* is propagated by conventional methods (seeding, sucker division, and grafting)^6–8^.

Unfortunately, the expansion of *R. coriaria* cultivation is presently restricted due to slow propagation, and because of seed dormancy, seed germination rate is very low whereby the sexual reproduction of the plants remains difficult. Propagation through new shoots from rhizomes and sucker division has a low reproduction rate^7,8^ while grafting is intricate and time-consuming^9^. So, alternative techniques such as micropropagation is immediately necessary to produce large numbers of rootstocks for *R. coriaria*. Micropropagation with high regeneration rate is a major vegetative multiplication method that could be used for the reproduction of uniform plants and providing frequent and abundant saplings in a short time throughout the year^10,11^.

Direct multiple shoot induction is the appropriate method for plantlet propagation from mature trees with a lower risk of genetic variability compared to other regeneration methods, and it is a more reliable for clonal propagation^12–15^.

Despite, its phytochemistry, pharmacological properties and industrial applications, few studies have been performed on the micropropagation of Sumac^16,17^. Several micropropagation procedures have been published for different species of the Anacardiaceae family such as *Pistacia* vera^18–23^. However, a general protocol has not been reported for efficient in vitro regeneration of this important species.

In micropropagation, several factors such as media and plant growth regulator (PGRs) composition may affect the results^24^. Several molecular methods are used for the assessment of genetic homogeneity and lack of somaclonal variation in tissue-derived plants including Restriction Fragment Length Polymorphism (RFLP), Amplified Fragment Length Polymorphism (AFLP), Random Amplified Polymorphic DNA (RAPD), Simple Sequence Repeat (SSR), Inter Simple Sequence Repeat (ISSR) and Sequence-related Amplified Polymorphism (SRAP)^25–27^. In this study, the effects of PGRs on all stages of in vitro propagation of *R. coriaria* i.e., establishment, proliferation and rooting were investigated to develop an efficient protocol for in vitro mass propagation of *R. coriaria*, and the genetic fidelity of the obtained plants was assessed.

## Materials and methods

Plant materials were collected from Aq Beraz, Hurand, Ahar County, East Azarbaijan province, Iran (39°00′39″N 47°23′34″E) in the early growing season (May 2018) of *R. coriaria*. The current year stem segments with axillary and lateral buds (2–3 cm length), were exposed to sterilization procedures^23,28^. The explants (2–3 cm segments with lateral buds) were washed thoroughly under running tap water for 25 min and then treated with few drops of tween-20 and 1 g/L of mancozeb (antifungal) for one minute with constant shaking by hand, followed by three successive washings with distilled water. Then, stem segments were disinfected for one minute with 70% ethanol, for 10 min in a solution of sodium hypochlorite (NaOCl 2.6% available chlorine), and were finally rinsed 3 times with sterile distilled water in a laminar airflow cabinet.

### Culture medium and growth conditions

The basal MS^29^ medium supplemented with 20 mg/L casein hydrolysate^30^ (Duchefa, Germany), 100 mg/L phloroglucinol^31^, 0.2% activated charcoal^23,32^ (used to reduce tissue oxidation due to phenolic compounds during explant establishment), 3% (w/v) sucrose and 7 g/L agar was used in this study. Different PGRs including 6-benzylamino purine (BAP) as cytokinin and indol-3-butyric acid (IBA) and naphthalene acetic acid (NAA) as auxin (Duchefa, Germany) are used at different concentrations (mg/L). The medium was adjusted to 5.8 ± 0.05 prior to autoclaving at 121 °C for 15 min. The cultures were incubated in phytotron chambers at 24 ± 2 °C under a 16/8-h (light/dark cycle) photoperiod provided by cool white fluorescent lights (1500–3000 Lx)^33^.

### PGRs treatments for establishment and shoots proliferation

The effect of different concentrations and combinations of PGRs in the full-strength modified MS medium on shoot bud initiation, multiple shoot induction, and shoot elongation were evaluated. The PGRs treatments tested for establishment and shoot proliferation are BAP at 0, 1, 2, and 3 mg/L with IBA at 0, 0.25, and 0.5 mg/L, in combination with constant 0.5 mg/L GA3 (Gibberellic acid) in MS medium. The percentage of explant establishment (established explants/explants × 100%) was recorded after three weeks, and shoot proliferation (shoots/explants) and shoot length (cm) was recorded after seven weeks.

### PGRs treatments for root induction

For rooting, IBA was used at 0, 0.5, 1.5, and 2 mg/L, combined with 0, 0.5, and 1 mg/L NAA in full and half-strength of MS medium. Individual propagated shootlets (3–4 cm) were separated and cultured in the root induction treatments. The percentage of rooting and average root numbers (roots/shoot) were observed and recorded after two weeks.

### Plantlets hardening

The rooted plantlets were obtained from the culture flasks after three weeks and washed carefully to remove any medium residue, then treated with 1 g/L Captan fungicide (AGROXIR, Iran) and potted into plastic cups in a mixture of peat moss and perlite (1:2 v/v), covered with transparent plastic cups to maintain high humidity. The plants transferred to greenhouse conditions with 25 ± 1 °C temperature and 75–85% relative humidity and preserved. After hardening for 21 days, transparent plastic bags were removed for further growth. Subsequently, the plants were transferred to plastic pots and the effect of different substrate compounds including peat moss (1v), perlite (2v) and soil (clay loam) (3v) and their compositions interaction evaluated on survival rate, plant height and root characteristics during the acclimatization process.

### Genetic uniformity assessment of micropropagated plants by ISSR

For genetic uniformity surveys, the genomic DNA was extracted by the cetyl trimethyl ammonium bromide (CTAB) method^34^ from fresh leaves of nine randomly selected in vitro grown plants and of the mother plant. The quality and concentration of DNA were measured by NanoDrop spectrophotometer (Nano Drop 1000, Thermo Scientific, USA). The ISSR analysis was performed using ten ISSR primers (Sina Clone, Iran), as used for the genetic fidelity test of *Crataegus* sp by ISSR in a previous study^12^. DNA amplification for ISSR markers was completed in 13.5 μL enclosing 6.25 μL PCR master mix (Sina Clone, Iran), 5.25 μL ddH2O, 0.5 μL template DNA (50–60 ng). ISSR amplification was attained in an Eppendorf thermal cycler (Eppendorf, USA) using the following program: 94 °C for 5 min in initial denaturation stage, 35 cycles of denaturation at 94 °C for 60 s, annealing at 55 °C for 45 s, and extension at 72 °C for 1 min with a final extension at 72 °C for 5 min. The PCR products were visualized by electrophoresis on 1.2% agarose gel in 1 × TBE buffer and stained with 7% ethidium bromide and photographed in Bio Doc Analyze Gel Documentation System (Biometra, USA). To estimate the length of the amplified products the 100 bp DNA ladder (Sina Clone, Iran) was used.

### Statistical analysis

A completely randomized design (CRD) of experiments with three replicats, was used. Each replicate included three glass jars (60 × 80 mm) containing four explants per glass. Data were analyzed statistically by SPSS software (version 16) followed by Duncan’s test, with *P* < 0.05; the results were represented as mean ± standard error of the three replicates.

## Results and discussion

### Explant establishment

**Figure 1:**
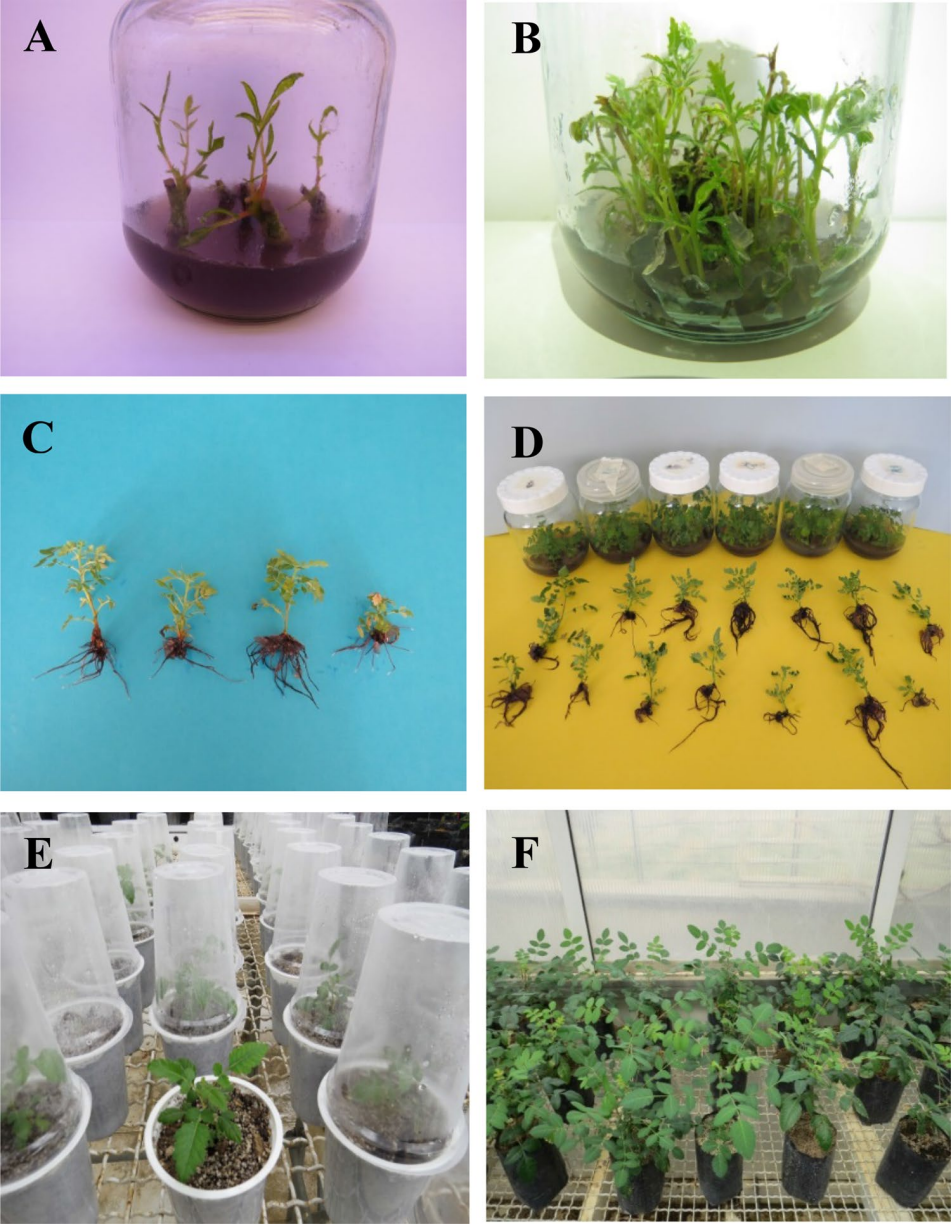
Different stages of micropropagation of *R. coriaria*; (**A**) establishment; (**B**) shoot multiplication; (**C**) rooting; (**D**) mass propagation; (**E**) acclimatization in plastic cups; (**F**) final adaptation in the greenhouse.

The results showed significant differences in the establishment rate among the treatment groups (Table 1). The explants started to respond 8–18 days after culture on MS medium supplemented with different concentrations and combinations of BAP and IBA (Table 2). The higher concentrations of BAP and IBA caused the earlier blooming of buds without necrosis (Fig. 1A). The highest average establishment (88.88 ± 1.01%) occurred on 3 mg/L BAP combined with 1 mg/L IBA (Table 2). Previous studies on in vitro propagation of *R. coriaria* found that MS medium supplemented with 1 mg/L BA + 0.5 mg/L IAA was best for callus induction and regeneration^16,17^. In this study, we regenerated new plantlets from lateral buds, which are more stable than plants raised from callus. The lowest establishment percentage (11.11 ± 0.80%) was observed in the hormone-free treatment.

**Table 1:** The effect of BAP and IBA treatment on the number of shoots per explant and shoot length (cm) of *R. coriaria.*

Source of variation	df	Mean squares (MS)		
		Establishment rate%	Shoot proliferation (shoots/explant)	Shoot length (cm)
BAP	3	5097.86**	105.61**	45.73**
IBA	3	2171.25**	15.48**	5.98**
IBA × BAP	9	391.92**	7.25**	3.27**
Error	30	1.35	0.47	1.02
Coeff var (C.V)	–	23.79	16.86	2.87

*; **Significant at the 0.05, 0.01 probability level respectively.

**Table 2:** The effect of BAP and IBA combinations on in vitro propagation of *R. coriaria.*

PGRs (mg/L)^a^		Establishment rate%^b^	Time of bud initiation	Shoot proliferation (shoots/explant)	Shoot length (cm)
BAP	IBA				
0	0.00	11.11 ± 0.80^g^	18th day	1.00 ± 0.06^h^	1.50 ± 0.03^e^
	0.25	11.11 ± 0.86^g^	18th day	1.00 ± 0.05^h^	2.00 ± 0.06^e^
	0.50	11.11 ± 0.76^g^	18th day	1.00 ± 0.04^h^	2.00 ± 0.05^e^
	1.00	22.22 ± 1.06^f^	18th day	1.00 ± 0.03^h^	2.00 ± 0.03^e^
1	0.00	22.22 ± 0.95^f^	16th day	1.2 ± 0.02^h^	2.50 ± 0.04^e^
	0.25	33.33 ± 0.56^e^	14th day	3.0 ± 0.10^f^	6.00 ± 0.45^b^
	0.50	33.33 ± 0.76^e^	14th day	3.5 ± 0.35^f^	6.00 ± 0.55^b^
	1.00	44.44 ± 0.46^e^	14th day	2.75 ± 0.5^g^	5.50 ± 1.00^b^
2	0.00	22.22 ± 1.02^f^	14th day	1.5 ± 0.15^h^	2.00 ± 0.25^c^
	0.25	55.55 ± 0.86^d^	12th day	8.50 ± 0.20^b^	3.50 ± 0.40^d^
	0.50	55.55 ± 0.77^d^	12th day	**12.30 ± 0.30**^**a**^	3.00 ± 0.35^d^
	1.00	77.77 ± 1.22^a^	8th day	7.30 ± 0.22^c^	4.00 ± 0.45^c^
3	0.00	33.33 ± 1.16^e^	12th day	1.85 ± 0.15^h^	2.30 ± 0.25^b^
	0.25	55.55 ± 0.55^d^	10th day	4.30 ± 0.45^e^	6.50 ± 0.30^b^
	0.50	66.66 ± 1.46^c^	8th day	5.20 ± 0.40^d^	5.50 ± 0.55^b^
	1.00	**88.88 ± 1.01**^**b**^	8th day	4.20 ± 0.30^e^	8.50 ± 0.75^a^

Different lower-case letters in each column indicate that these values are statistically different at *P* ≤ 0.05 according to Duncan’s Multiple Range Test.

^a^All media also containing 0.5 mg/L GA3 and 0.2% activated charcoal.

^b^ Each value represents the mean ± Standard Error of three replicates.

The bold values indicates the best treatment for establishment and prolifration.

### Shoot proliferation

The growth regulators are fundamental compounds in plant tissue culture media that regulate diverse physiological and developmental processes in plants such as cell elongation (stem, root), leaf expansion, photomorphogenesis^35^. One of the most important groups of PGRs are the cytokinins (CKs) which play a crucial role in regeneration and shoots proliferation^36^. The most useful and universally used cytokinin for in vitro culture is BAP as it can metabolize immediately in plant tissues^37^, and it was therefore used for shoots proliferation and shoot elongation in this study. Following the establishment stage, the shoots longer than 2 cm were selected and then transferred onto the shoot proliferation treatments. Based on variance analysis results, there was a significant difference in shoot proliferation and shoot elongation among the different treatment groups (Table 1). Our results showed MS medium fortified with PGRs provided better conditions for establishment percentage, shoot proliferation and elongation than MS without PGRs (Table 2). The shoot proliferation rate was enhanced on the intermediate concentrations of BAP and IBA. On the contrary, a decrease in the multiplication rate observed with lower concentrations of BAP and IBA. The highest proliferation rate (12.30 ± 0.30) was achieved on the MS medium supplemented with 2 mg/L BAP and 0.5 mg/L IBA (Fig. 1B). The maximum bud growth and shoot length (≧ 8 cm), were observed on 3 mg/L BAP combined with 1.0 mg/L IBA (Table 2). The rate of proliferation is the most important phase in micropropagation, and MS medium with 2 mg/L BAP and 0.5 mg/L IBA was the best treatment to get the highest number of shoots with a suitable length for rooting (Fig. 1B). IBA is critical in cell division and BAP is important in cell expansion, so it is crucial to retain an optimal BAP and IBA ratio for shoot proliferation and elongation^38^. Increasing the BAP concentration to induce a higher shoot proliferation caused a shortened shoot length due to an increased competition among buds^12^, while lower BAP concentrations shoots had fewer lateral branches, and exhibited a better elongation due to the reduced competition among the shoots.

### Rooting and hardening

Root induction in in vitro as ex vitro is one of the important limiting factors in plant propagation. In this study, rooting occurred two weeks after culture and significant differences were observed in the percentage of rooting and root numbers among the treatments applied (Table 3). IBA and NAA, alone or together, were used to promote root formation. The treatment with IBA was more effective than NAA, in agreement with previous study by Safarnejad et al.^17^, who attained highest rooting of shoots on MS medium supplemented with 1 mg/l IBA. In the other study, liquid and solid media for rooting were tested and the highest rooting percentage (44.83%) was observed on solid media^16^. Increasing IBA levels (up to 1 mg/L), increased the frequency of root induction but this gradually decreased when 2 mg/L IBA was used (Table 4, Fig. 1C), perhaps due to the endogenous auxin level. The highest rooting percentage and average root numbers were achieved in a half-strength MS fortified with 1 mg/L IBA and 0.5 mg/L NAA after two weeks of culture. Moreover, addition of IBA or NAA alone was not satisfactory for rooting. IBA at 1 mg/L combined with 0.5 mg/L NAA resulted in the highest rooting (100%), in line with previous reports showing that medium strength, medium type, concentration and duration of auxin treatment are all essential for root induction^39^. Full- or half-strength MS media without any plant growth regulators failed to induce rooting of the propagated shoots. A reduced rooting percentage (11.11 ± 0.5%) was achieved in the hormone-free MS medium (control). The same observation was true for the optimum root number (Fig. 1D). The use of longer shoots (> 6 cm) had a positive effect on root induction and acclimatization.

**Table 3:** Analysis of variance of IBA and NAA treatments on the rooting of *R. coriaria.*

Source of variation	df	percentage of rooting	Number of roots per rooted shoot
IBA	2	5275.48**	11.73**
NAA	3	1849.31**	2.35**
IBA × NAA	6	463.67**	1.62**
Error	22	20.76	0.40
Coeff var	–	9.11	19.86

*; **Significant at the 0.05, 0.01 probability level respectively.

**Table 4:** Effects of auxins (IBA, NAA and IBA * NAA) on the rooting of *R. coriaria.*

MS medium	PGRs (mg/L)		Percentage of rooting	Root number
	IBA	NAA		
Full strength	0	0.00	11.11 ± 0.5^f^	1.2 ± 0.30^i^
		0.25	22.22 ± 0.5^e^	1.5 ± 0.10^g^
		0.50	22.22 ± 0.6^e^	1.5 ± 0.20^g^
		1.00	22.22 ± 0.3^e^	1.6 ± 0.30^f^
	1	0.00	22.22 ± 0.5^e^	1.3 ± 0.10^h^
		0.25	44.44 ± 0.5^c^	2.2 ± 0.25b^c^
		0.50	66.66 ± 0.3^a^	3.5 ± 0.11^a^
		1.00	33.33 ± 0.4^d^	2.4 ± 0.20^b^
	2	0.00	33.33 ± 0.3^d^	1.5 ± 0.35^g^
		0.25	44.44 ± 0.5^c^	1.8 ± 0.20^d^
		0.50	55.55 ± 1.0^b^	1.7 ± 0.40^ed^
		1.00	55.55 ± 1.3^b^	1.5 ± 0.20^g^
Half strength	0	0.00	11.11 ± 1.2^g^	1.2 ± 0.10^e^
		0.25	33.33 ± 0.3^f^	2.5 ± 0.35^d^
		0.50	33.33 ± 0.5^f^	2.5 ± 0.11^d^
		1.00	33.33 ± 0.4^f^	2.7 ± 0.20^d^
	1	0.00	33.33 ± 0.2^f^	2.7 ± 0.30^d^
		0.25	77.77 ± 1.0^b^	4.2 ± 0.50^b^
		0.50	**100.00 ± 0.0**^**a**^	**5.5 ± 0.20**^**a**^
		1.00	66.66 ± 0.5^c^	4.4 ± 0.30^b^
	2	0.00	44.44 ± 0.3^e^	3.5 ± 0.25^c^
		0.25	55.55 ± 0.5^d^	3.1 ± 0.50^c^
		0.50	66.66 ± 0.4^c^	3.2 ± 0.40^c^
		1.00	55.55 ± 0.35^d^	3.0 ± 0.20^c^

Each value represents the mean ± SE of three replicates. Different lowercase letters in the same column indicated the significant difference at *P* ≤ 0.05 (Duncan’s multiple range test).

The bold values indicates the best treatment for rooting.

One of the usual problems of plant propagation of in vitro methods is the acclimatization to ex vitro conditions, which adds to a high mortality of plantlets caused by fungal contamination to which in vitro raised plants are very vulnerable^40^. Thus, 1 g/L Captan (fungicide) was used to control fungal contamination. The results of hardening showed significant differences in the acclimatization of plantlets among the applied planting bed composition types (Table 5, Fig. 1E). Thus, the highest plantlet height, vigor percentage, root number, and root length were observed on perlite, peat moss, and soil (clay loam) mixture at a ratio of 2:1:3 (v:v:v) (Table 5). Then, following Captan treatment, a survival rate of about 87% of plantlets was reached (Fig. 1F).

**Table 5:** The effects of planting bed compositions (perlite, peat moss, and soil) on the acclimatization of *R. coriaria.*

Planting bed composition	Plantlets height	Leaf numbers	Vigourity percentage	Root numbers	Root length (cm)
Perlite	2.5 ± 0.5^g^	2 ± 1.0^e^	20 ± 1.5^g^	3.5 ± 0.5^f^	2.5 ± 0.6^f^
Peat moss	5.5 ± 0.5^f^	5 ± 1.5^d^	30 ± 2.5^f^	3.0 ± 1.5^e^	3.5 ± 0.5e
Soil (clay loam)	15 ± 2.5^c^	8 ± 2.0^c^	65 ± 1.5^c^	6.5 ± 2.5^c^	8.5 ± 1.5^c^
Perlite + peat moss (2:1)	7.5 ± 0.5^e^	5 ± 0.65^d^	40 ± 0.5^e^	4.5 ± 0.5e	6.5 ± 1.0^d^
Perlite + soil (2:3)	20 ± 1.0^b^	15 ± 1.5^b^	75 ± 1.5^b^	7.5 ± 1.0^b^	14.0 ± 0.5^b^
Peat moss + soil (1:3)	12 ± 0.5^d^	5 ± 1.0^d^	55 ± 3.5^d^	5.5 ± 1.5^d^	4.5 ± 1.5^d^
Perlite + peat moss + soil (2:1:3)	30 ± 1.5^a^	20 ± 2.0^a^	87 ± 0.5^a^	15.0 ± 1.5^a^	20.0 ± 20^a^

Each value represents the mean ± SE of three replicates. Different lowercase letters in the same column indicated the significant difference at *P* ≤ 0.05; Different uppercase letters in the same column indicated the significant difference at *P* ≤ 0.01 (Duncan’s multiple range tests).

### Assessment of genetic stability of micropropagated plantlets by ISSR

**Figure 2:**
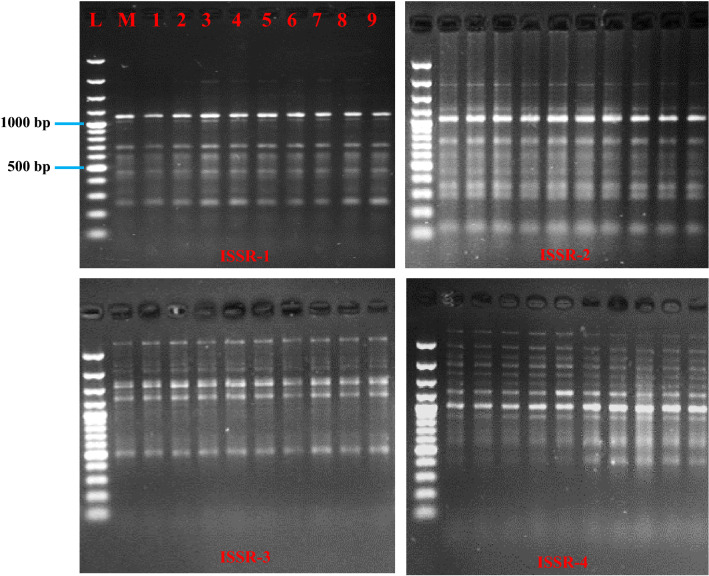
ISSR profiles generated by PCR amplification. Lane L: molecular marker (100 bp); lane M: mother plant; lane 1–9 in vitro raised plants.

Genetic fidelity of micropropagated plants must be ascertained for preservation of genetic uniformity before confirming the success of a micropropagation protocol. To study genetic stability, we have compared the fragments amplified using a set of ten ISSR primers in the micropropagated plantlets of *R. coriaria* with the fragments obtained in the mother plants. ISSR is cost-effective, requires low amounts of DNA and especially, semi-arbitrary and medium to highly reproducible. ISSR markers are segregated as dominant markers and they are easy to handle and more reproducibly than other markers^25,41^. All ten ISSR primers produced clear and reproducible amplification products (supplemental section). They generated 74 clear bands in total, ranging from 100 to 3000 bp in size (Table 6, Fig. 2). We have not found variation in the fragments amplified from micropropagated plantlets compared with the mother plant, confirming that they are suitable for large-scale and true-to-type micropropagation of *R. coriaria*. Our findings also confirmed other previous reports where plants micropropageted through organized cultures, especially shoot tips and axillary buds, preserve genetic stability^42,43^.

**Table 6:** Details of ISSR primers and total number of bands and size range of amplified fragments generated by ISSR makers in *R. coriaria.*

Primer code	Primer sequence (5–3)	Annealing temperature	Total bands scored	Size range of amplified fragments (bp)
ISSR-1	(GGGGT)_3_	52	9	250–2000
ISSR-2	(AG)_8_CC	52	9	150–2000
ISSR-3	(TCC)_5_TG	54	7	500–3000
ISSR-4	(AC)_8_YG	54	12	400–3000
ISSR-5	(GA)_8_GCC	54	3	500–1500
ISSR-6	(GT)_8_T	55	4	250–2000
ISSR-7	(GA)_8_A	55	7	500–3000
ISSR-8	(AG)_8_T	55	9	150–2000
ISSR-9	(GA)_8_T	55	5	300–1000
ISSR-10	CCC (GT)_7_	55	9	100–2000

## Conclusion

In conclusion, we achieved a high shoot multiplication rate and rooting as a reliable and reproducible protocol for micropropagation of *Rhus coriaria* L. Our results also indicated that the maximum rate of proliferation (12.3 ± 0.30) was achieved in MS medium fortified with 2 mg/L BAP, 0.5 mg/L IBA and 0.5 mg/L GA3. ½ MS medium supplemented with 1 mg/L IBA combined with 0.5 mg/L NAA resulted in 100% rooting. The ISSR analysis confirmed the reliability of this protocol for the efficient large-scale micropropagation of *Rhus coriaria.*

## Supplementary Information

Supplementary Information.
