# Supplementary material for: Establishment of an efficient in vitro propagation protocol for Sumac (Rhus coriaria L.) and confirmation of the genetic homogeneity
Source: Sci Rep. 2021 Jan 8;11:173. doi: 10.1038/s41598-020-80550-4 (PMC7794432; doi:10.1038/s41598-020-80550-4)
Supplement: Supplementary file 1 — Supplementary Information. [file 41598_2020_80550_MOESM1_ESM.pdf]

Establishment of an efficient *in vitro* propagation protocol for Somac (*Rhus coriaria* L.) and confirmation of the genetic homogeneity

Authors: Saleh Amiri and Reza Mohammadi*

Branch for Northwest & West region, Agricultural Biotechnology Research Institute of Iran (ABRII), Agricultural Research, Education and Extension Organization (AREEO), Tabriz, Iran.

*Corresponding author: r.mohammadi@abrii.ac.ir

Supplementary Information file 1. Original ISSR profiles generated by PCR amplification.

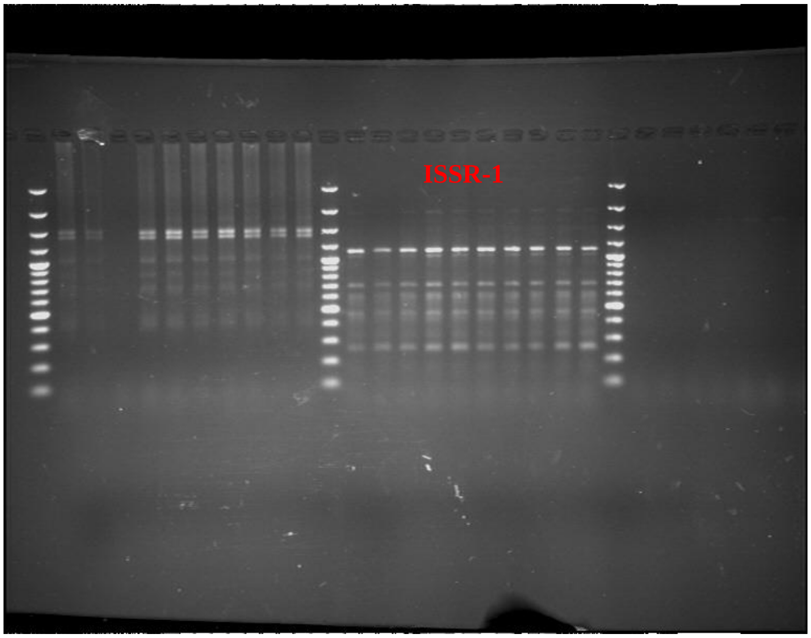

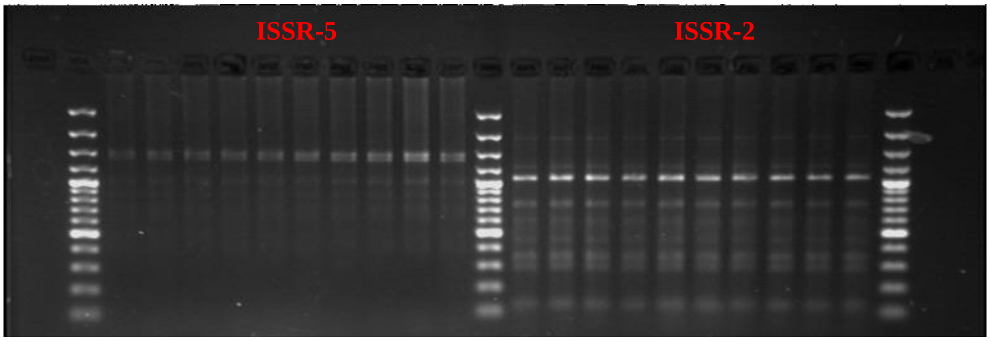

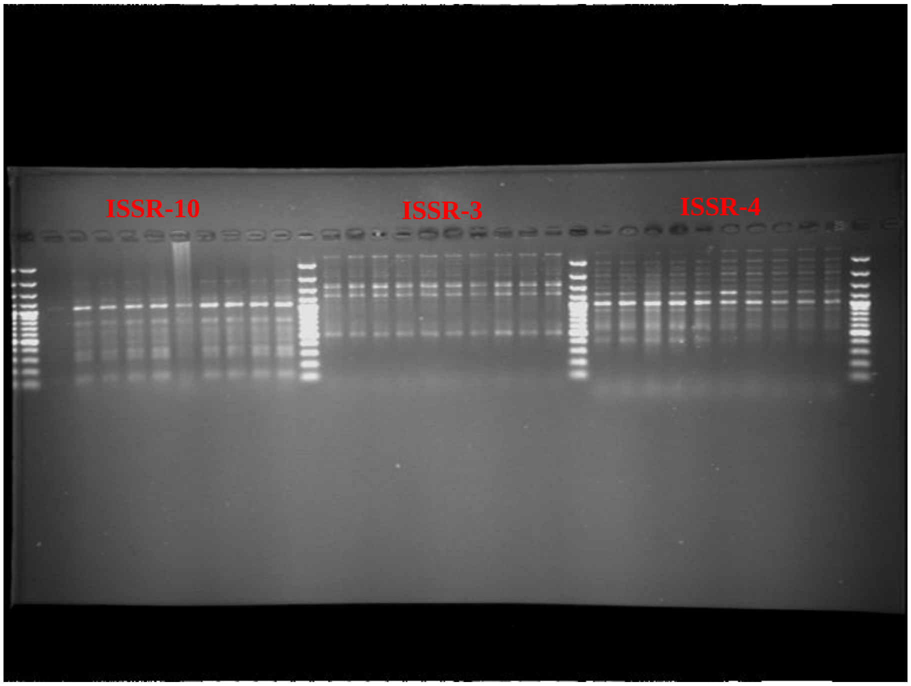

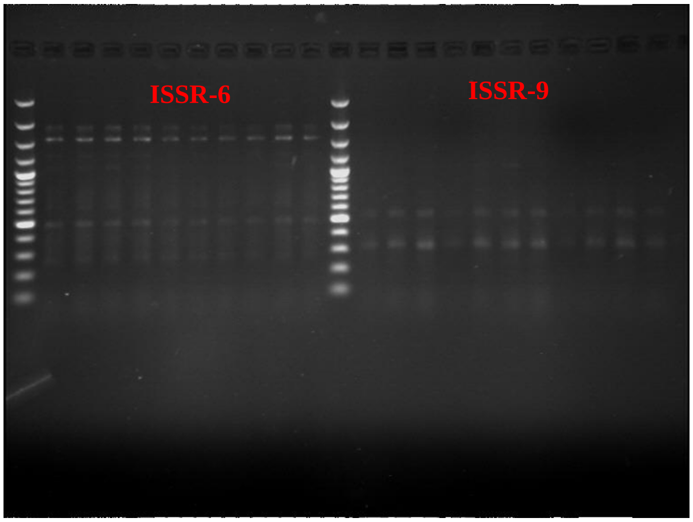
ISSR-6
ISSR-9

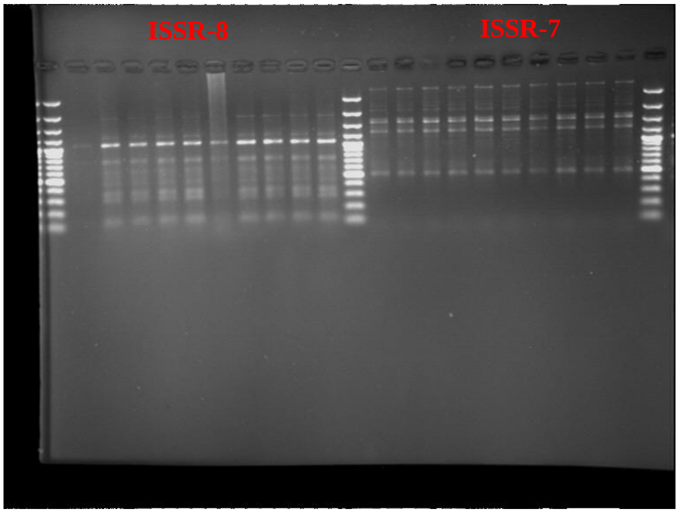
ISSR-8
ISSR-7
